# Reflections on clinical practice whilst developing a portfolio of evidence: Perceptions of undergraduate nursing students in the Western Cape, South Africa

**DOI:** 10.4102/curationis.v38i2.1502

**Published:** 2015-12-09

**Authors:** Victoire Ticha, Lorraine P. Fakude

**Affiliations:** 1 School of Nursing, University of the Western Cape, South Africa

## Abstract

**Background:**

In order to develop clinical judgement, nurses should be encouraged to become analytical and critical thinkers. Development of a portfolio of evidence (PoE) of reflection on clinical experiences is one of the strategies that can be used to enhance analytical and critical thinking amongst nursing students. Students’ perceptions of the process are important in order to encourage their reflective practice. PoE compilation at a school of nursing at a university in the Western Cape includes evidence of students’ clinical learning which they present in a portfolio. The students are expected to reflect on their clinical learning experiences and include these reflections in their portfolios.

**Objective:**

To describe the perceptions of fourth-year nursing students regarding reflective practice whilst compiling their PoEs.

**Method:**

A qualitative design was used to explore the perceptions of registered fourth-year nursing students with regard to their reflective practice whilst compiling their PoEs. Purposive sampling was used for selection of participants. Three focus group discussions were held, each consisting of six to eight participants. Data saturation was reached during the third meeting. Tesch’s method of data analysis was used.

**Results:**

Findings revealed that reflection enabled the learners to gain experience and identify challenges related to the expected events and tasks carried out at the hospitals and in the classroom whilst developing their PoE.

**Conclusion:**

The compilation of a PoE was a good teaching and learning strategy, and the skills, experience and knowledge that the participants in this study acquired boosted their self-esteem, confidence and critical thinking. Reflection also assisted in self-directed learning.

## Introduction

Nurses play a pivotal role in the South African health system, as they are usually one of the first points of call for patients. The nature and challenges of the nursing profession require nurses to act autonomously whilst at the same time making appropriate clinical judgements. With this perspective, educators are tasked with the challenge of assisting nurses to make competent clinical decisions in everyday situations (Chirema 2007:192–202).

The accomplishment of safe clinical judgements improves nurses’ ability to become analytical and critical thinkers. One of the tools that nurse educators can use to enhance analytical and critical thinking is to encourage students to reflect whilst they are engaging in the development of a portfolio of evidence (PoE). Reflective practice has been used to bridge the gap between nursing theory and practice and to articulate and develop nursing knowledge embedded in practice (Chong 2009:111). The undergraduate nursing students are expected to reflect on practice events and skills that they encounter during their learning process in the classroom and in clinical settings.

### Background

A PoE is a form of assessment that provides samples of the students’ work which reflect their development over time. By reflecting on their own learning (self-assessment), students begin to identify the strengths and weaknesses in their work. These weaknesses then become improvement goals. Norris and Gimber (2013:17–18) carried out a study at LaGuardia Community College in New York, where e-portfolios were used to record the reflective practice of the nursing students; results revealed that the students’ performance improved. An e-portfolio comprises a collection of student assignments that are presented digitally, similar to a portfolio that professionals use for displaying their work. The main distinguishing feature of an e-portfolio is the digital portrayal of student work, in contrast to a traditional paper-based portfolio. In a study by Green, Wyllie and Jackson (2013) e-portfolios provided a means for nurses to record and provide evidence of skills, achievements, experience, professional development, and continual learning. The e-portfolios were not only benefitting the nursing students, but the information was also available to registration boards, employers, managers, and peers.

At this university in the Western Cape, the undergraduate nursing students compile a paper-based PoE in which they produce examples of clinical learning opportunities they reflected upon. It is submitted at the end of every semester, and forms an integral part of the formative assessment of a module. In this PoE, the nursing students provide evidence of core clinical assessments, clinical tutorials, records of hours of clinical learning, a progress report written by a professional nurse at the clinical placement area where the student was allocated to during that academic period (month/term), self-directed learning records about learning activities in the clinical skills laboratories, peer assessment tools, and any incidents which they experienced in the course of compilation. These are the pieces of evidence that the nursing students reflect on as they develop their PoE. The nursing students reflected by writing down their perceptions, which included their experiences when collecting the abovementioned pieces of evidence and any other incidents they encountered in the course of compiling their PoE.

This research study at the school of nursing in the Western Cape explored the perceptions of the students on their clinical experiences during their PoE compilation as evidence of their learning. Students selected evidence of learning activities on which they reflected. Ninety percent of the evidence is purely of a clinically nature, and, therefore, 90% of the presented information in this study relates to the clinical setting.

### Purpose of the study

This study explored the perceptions of fourth-year nursing students in relation to reflective practice during the development of their PoEs. The research question was as follows: ‘What are the perceptions of fourth-year nursing students in relation to reflective practice whilst compiling their PoEs?’

### Significance of the study

The findings of this study can be used to modify the curriculum for nursing students and may also be used as a point of departure for future research.

Students will indirectly benefit from this study as their clinical learning experiences during compiling a PoE will be made known to the nursing education community, therefore contributing to the knowledge base and potentially positively impacting nursing practice.

Through continuous reflection the students will also be able to identify their weaknesses and strengths. Through self-assessment they will be able to identify where to improve.

### Literature review

Jensen and Joy (2005:139) say reflection has been advocated as a way of bridging the gap between theory and practice. Reflective practice is one of the ways in which educators develop thoughtful, intelligent and careful practice. According to Ryan (2011:84), a newly revised curriculum was the impetus for implementing the use of portfolio development at the Adelphi University School of Nursing in New York. The portfolio was incorporated as it was viewed as a vehicle for self-reflection and a way for the nursing students to document academic and professional accomplishments whilst progressing during their nursing programme. Its use in a medium size group of nursing students was assessed, and Ryan found that the portfolio provided an alternative/adjunct method for assessing nursing students’ performance whilst developing advanced nursing practice skills, achieving role development and attaining established graduate nursing programme outcomes. Also, the students were convinced that using a portfolio kept them academically focused.

As mentioned earlier, Norris and Gimber (2013:17–18) state that an e-portfolio (digital portrayal of students’ learning activities) assists nursing students with implementing reflective practice. Green *et al*. (2013) outline how e-portfolio development has been used at the University of Technology in Australia for a number of years to encourage reflective practice of nursing students. The students collect and select appropriate materials to create a body of work that is representative of their learning for the duration of their education. The study further explains that an e-portfolio can represent an authentic means of assessing cognitive, reflective and affective skills, whilst equipping nurses with an effective tool to record and provide evidence of skills, achievements, experience, professional development, and continual learning (Green *et al*. 2013). Such evidence of skills not only benefits the nursing students as the the information is also available for scrutiny by registration boards, employers, managers, and peers.

According to Chang *et al*. (2013:217), e-portfolios simply reinforce the knowledge management that universities have been involved in ever since their establishment. They showed that compilation of an e-portfolio significantly facilitated performance of the knowledge management process.

In a study carried out at the University of Pretoria, Maree (2007:210) emphasises that reflective practice is an approach which meets the demands of a specific context by using more than just rational and evidence-based knowledge and skills. It includes experience and personal growth, based on the underlying processes of reflective practice and the hierarchy of competencies. These practices have positive outcomes for the nursing students and the community. Reflective practice has been included in their model for reflective training of neonatal nurses.

The literature provides evidence that reflective practice is implemented internationally. Also, reflective practice may be implemented through electronic journaling and compiling e-portfolios. Reflective practice not only benefits the teaching and learning process of nursing students, but students in other educational disciplines too. Maintaining a portfolio offers a significant opportunity for reflection on continual development. Joyce (2005) mentioned that a:

… portfolio of work that incorporates self-reflection supports learning if the developmental nature of the portfolio process is sustained and provides opportunities for students to self-evaluate their own growth. (p. 459)

In a study carried out by Jeggels, Traut and Africa (2013), participants had to compile a PoE for assessment purposes. The course evaluations and reflective journaling by participants revealed that all of the desired outcomes of the course had been achieved. The managers who nominated their staff to participate in the continual education offering also provided positive feedback. The above presented information encouraged the researcher to explore the perceptions of fourth-year nursing students at a university regarding their reflective practice whilst they were developing their PoEs.

## Research design and methodology

A qualitative approach and exploratory design was used to investigate the general perceptions of fourth-year nursing students regarding reflective practice that they implemented whilst compiling their PoEs for the purpose of formative assessment.

This study was conducted at a school of nursing in the Western Cape. Data collection was arranged with the lecturers teaching in the first year of the Bachelor of Nursing programme and the clinical coordinators teaching first year students in order to access the potential participants.

### Population and sampling

The population comprised all registered fourth-year Bachelor of Nursing students (*n* = 172) at the university. Fourth-year level nursing students were chosen because they had completed their fourth-year first semester PoE compilation, and were familiar with the process of portfolio compilation. Hence they were willing to share their experiences because they understood the research objective, which was to describe the perceptions of fourth-year nursing students about the reflective practice in PoE and its educational characteristics.

The researcher conducted convenient sampling of fourth-year nursing students as they had a better understanding and insight into reflective practice for the purpose of compiling their PoEs at the end of every semester. Also, they had been engaged in this learning activity for a longer period of time compared with students at other levels in the programme.

According to Onwuegbuzie and Leech (2007:242) a sample in qualitative research should neither be too large to obfuscate the extraction of thick, rich data nor too small for achieving data saturation. The inclusion criteria were fourth-year nursing students who had completed their first semester portfolios and were willing to share their perceptions and experiences of incidents encountered in the course of developing the PoE. The sample constituted 21 students who volunteered to participate in the study.

### Data collection

Three focus group discussions were conducted with eight, seven and six participants respectively. According to Terre Blanche *et al*. (2006:304) most focus groups are composed of 6 to 12 people.

Gibbs’s Reflective Cycle (1988) was used as a framework to guide data collection and analysis. The cycle involves six steps with the corresponding questions:

- Description of the situation/incident: ‘What happened?’
- Feelings: ‘What were you thinking and feeling?’
- Evaluation: ‘What was good and/or bad about the experience’?
- Analysis: ‘What sense can you make of the situation?’
- Conclusion: ‘What else could you have done?’
- Action Plan: ‘If the incident arose again, what would you do?’

The following opening question was asked: ‘Please describe your perceptions on reflective practice in your portfolios of evidence development at the school of nursing’. Other follow-up questions were asked to gain more insight into the research topic. The focus group discussions (FGDs) were voice recorded with the permission of the students, and handwritten notes were captured because the researcher did not want to miss any information that was discussed or demonstrated. Non-verbal communication, such as body gestures and facial expressions, was taken into consideration. The participants’ tone of voice and body language were also noted by the researcher.

Data were collected until data saturation was reached. By the second FGD the researcher noticed repetition of information (data saturation and patterns), with the result that the third FGD became a confirmatory session.

## Results and discussion

Two themes emerged from the data: challenges related to collecting evidence for PoE development, and students’ perceptions of development of PoE. Each theme had sub-themes, as shown in Table 1.

**TABLE 1:** Themes and sub-themes that emerged from the data.

Themes	Sub-themes
Challenges related to collecting evidence for PoE development	Racism Discrimination Professionalism Limited time Interpersonal relationships
Students’ perceptions of development of PoE	Skills enhancement Critical thinking and boosting of confidence

PoE, portfolio of evidence.

## Theme 1: Challenges related to collecting evidence for portfolio of evidence development

### Racism

The International Council of Nurses Code of Ethics states that:

Inherent in nursing is respect for human rights, including cultural rights, the right to life and choice, to dignity and to be treated with respect. Nursing care is respectful of and unrestricted by considerations of age, colour, creed, culture, disability or illness, gender, sexual orientation, nationality, politics, race or social status. (International Council of Nurses [ICN] 2006:1)

Essentially this means that all people should be treated equally. This is also reflected in the South African Nursing Code of Ethics that reminds nurse practitioners to carry out their responsibilities with:

… the required respect for human rights, which include cultural rights, the right to life, choice and dignity without consideration of age, colour, creed, culture, disability or illness, gender, sexual orientation, nationality, politics, race or social status. (South African Nursing Council 2013:3)

In this study racism was experienced by one participant in focus group 1 (FG1, P3) who felt unfairly treated by a professional nurse (PN) in a Midwifery Obstetric Unit (MOU). The PN expected the student to assist a patient who was in labour without putting on gloves, therefore putting the student nurse at risk of cross-infection. The participant (FG1) was assertive and indicated that:

‘I do not know the patient’s HIV status … the PN continued to shout at me in front of other patients.’ (P5)

The participant further said:

‘“I felt that was racism and victimisation, since I am black”. The incident affected that participant negatively to the point that the PN adjusted her assessment downwards on her progress report.’ (P5)

According to Jeggels *et al*. (2013), nursing students are under the supervision of clinical supervisors employed by a university for a limited period whilst being placed in the service unit. For the remainder of the time, the students are supervised by the PNs in the unit. This is linked to students’ experiences, in that the PN in this case failed to carry out part of her duties, which was student supervision and training. Other participants in FG1 and FG2 also indicated that racism seemed to be a problem amongst various racial groups in nursing; they felt that students were not treated equally on the basis of race.

### Discrimination

According to Bosher (2008:34–35) nursing students persistence in college and nursing programmes is a complex, multifaceted issue influenced by academic performance, support, discrimination and their ethnic culture; these influence nursing education.

One of the participants in FG2 said:

‘discrimination existed between students from their university and students from other higher education institutions (HEIs) who were placed at the same clinical facility.’ (P4)

For example, when a university student was placed in a ward with trained staff from another HEI, there seemed to be tension and conflict. The PNs who obtained their professional qualification from a higher education institution different to that of the student would not expose the university student to a learning opportunity, but would pay more attention to nursing students from the HEI at which they had trained. The student nurse on the other hand indicated that the PN who qualified at their university did not discriminate, and said that when they:

‘… work with PNs [*from this university*] I do not see any form of discrimination in the ward as students from both institutions are treated equally.’ (FG2, P4).

This participant in FG2 reflected that, because of the discrimination experienced in the ward, she felt belittled during clinical placement; this made her feel unhappy and took away her zeal to work.

Likupe and Archibong (2013:227–229) explored black African nurses’ experiences of equal opportunities, racism, and discrimination in four National Health Service (NHS) trusts in North East England. Thirty nurses from sub-Saharan countries working in four NHS trusts were interviewed between 2006 and 2008 using semi-structured interviews and FGDs to gain insight into their experiences. The study suggests that black African nurses experience severe discrimination and racism from white colleagues and nurses from other countries, managers, and patients and their relatives, and of opportunities in their places of work.

In the current study a participant said:

‘“There exists [*sic*] discrimination [*of*] nursing students by the … PNs”. She further reported that as a result of the discrimination they experienced they did not learn enough to meet their clinical objectives and did not acquire the necessary evidence for the PoE, and felt belittled while on duty. They felt demoralised during the clinical placement, since they were not given the expected learning opportunities by the permanent staff.’ (FG2, P3)

One of the participants in FG1 said:

‘she sometimes considered clinical placements a waste of time and attended simply to record her required number of clinical hours.’ (P6)

The FG1 participants felt that university students were not treated well because PNs preferred students from other HEIs, who were placed for a longer period, for example, three months. The students from the university in this study visited the clinical facilities once or twice a week and therefore did not have sufficient time to develop a relationship with the patients and staff, as indicated by a participant:

‘Students might not have sufficient time to create a bond/relationship with the patients and staff.’ (FG2, P3)

### Professionalism

According to Van der Colff and Rothmann (2009:10) professionalism in nursing includes a sense of significance, enthusiasm, inspiration, pride and challenge, whilst absorption is characterised by full concentration on and engrossment in one’s work, and finding it difficult to detach oneself from work.

The Code of Ethics for Nursing Practitioners in South Africa, Article 3.3 (South African Nursing Council 2013), addresses the requirement of beneficence. Nurses are obliged to do well, choose the best option of care under given circumstances, and act kindly at all times. These obligations are an expression of compliance with the imperative duty of care during professional practice. A participant in FG1 reflected on her satisfying experience in the hospitals, saying:

‘Just seeing the patients’ faces is good for me.’ (P3)

The researcher perceived that as a sign of professionalism and caring as a nurse.

According to Clarke *et al*. (2012:270), verbal abuse appears to be the most predominant form of bullying experienced by nurses as well as nursing students. In the current study the actions of the same PN at the MOU left the student feeling that she had experienced verbal, emotional, and psychological abuse:

‘The PN continued to shout at me in front of other patients.’ (FG1, P3)

Discrimination is likely to be a precursor to abuse. On the other hand, one of the participants in FG2 experienced that some operational managers (OPMs) made provision to assist the student nurses with meeting some of their clinical objectives. This participant provided the example of inserting a urinary catheter and catheter care that had occurred during her first year of training:

‘The OPM took time and explained so well, thereafter my clinical book was signed off by her.’ (FG2, P4)

This quotation is congruent to the requirements of the *Nursing Act* (Act No. 33 of 2005, Chapter 2), which indicates that the PN is a person who is qualified and competent to independently practice comprehensive nursing care in the manner and to the prescribed standard, and who is capable of assuming responsibility and accountability for such practice. The OPM is initially registered as a PN and then promoted to the higher position according to experience.

### Limited time for completing a portfolio of evidence

The honesty of entries affects the validity and credibility of the PoE as an assessment tool. Students complained that it took them a prolonged period to compile their portfolios for assessment. Joyce (2005:459) also mentioned that portfolios have been criticised for the amount of time taken to complete and assess them.

A participant in FG2 said:

‘Time in compiling the PoE has always been limited.’ (P3)

FG2 participants further supported this point of view by adding that it took substantial time, as much had to be included in the PoE. Pacquiao (2007:33) is adamant that if time and appropriate supervision is provided to nursing students, the students would feel more motivated about this learning activity. A participant in FG3 reflected that he found the completion of his PoE difficult during the first and second years of study, because:

‘Some of the clinical supervisors did not demonstrate skills well and created a tense environment so I was most of the time scared to ask questions and I know that I am not fluent in speaking the English language.’ (P4)

However, he managed to pass on the grounds of self-directed learning at the skills laboratory and by consulting his class lecturers and colleagues for clarification.

The researcher realised that poor communication and interpersonal relationships existed between the students and clinical supervisors. Under these circumstances teaching and learning only took place partially, owing to the tense learning environment. It appears that the preceptors (clinical supervisors) did not really provide professional guidance to this student.

Interpersonal relationships are emphasised by Jeggels *et al*. (2013), with the definition of a preceptor as a competent practitioner who provides professional guidance to students allocated to a service setting for a certain period of time (Yonge *et al*. 2007:9, in Jeggels *et al*. 2013). Support from preceptors enables nursing students to apply knowledge and skills in the clinical setting to facilitate the transformation from novice to expert. Preceptorship refers to ‘… an individualized period of support under guidance of an experienced clinical practitioner which attempts to ease transition into professional practice or socialization into a new role’ (United Kingdom Department of Health 2009:11, in Jeggels *et al*. 2013).

As an educator it is important to obtain feedback at the end of each and every educational session with learners. One of the ways to solicit feedback is to ask learners at the end of accession whether they have any questions or comments, or need any clarifications. This feedback enables the educator to identify issues or challenges related to the students’ learning.

## Theme 2: Students’ perceptions of portfolio of evidence development

Timmins and Dunne (2009) suggest that a portfolio attests to achievement and professional development by providing a critical analysis of its content. Similarly, it is suggested that a portfolio should at least illustrate a student’s ability to think critically, perform appropriate therapeutic nursing interventions, communicate effectively, and ultimately integrate theory and practice. In the context of this study those suggestions referred to the outstanding knowledge, skills and experience that some of the participants gained during the process of compiling their PoEs.

According to *Cambridge Dictionaries* Online, positivity is the quality of having a positive attitude. Some of the participants experienced a positive attitude whilst compiling their PoEs; ultimately that attitude enhanced skills such as organisation and administration. As one participant from FG1 reflected:

‘The skills laboratory was good as learning took place through incorporating theory into practice. Each session at the skills laboratory made us have that feeling of the hospital, hence boosting their confidence and behaviour.’ (P5)

Participants mentioned that they enjoyed practising and gaining experience in clinical skills such as bed making, bed bathing, baby bathing, administration of oral medication, abdominal palpations, and delivery of new born babies, removal of clips and sutures, and history taking. They also mentioned that practising some of the skills on the simulated patients minimised the feeling of fear and anxiety.

The positive experiences of participants included direct participation and taking ownership in the development of their PoEs, which boosted their learning process. For some the compilation of a PoE went well, whilst others struggled.

### Skills enhancement, critical thinking and boosting of confidence

This sub-theme refers to the knowledge and skills that some of the participants gained during the process of compiling their PoEs. One of the few studies on critical thinking in nursing education was carried out by Mangena and Chabeli in South Africa. According to Mangena and Chabeli (2005:292), the goal of critical thinking is to develop learners who are fair-minded, objective, as well as committed to clarity and the ever-changing and increasingly complex state of knowledge development, which demands higher order thinking of nursing students. As one participant of FG3 stated:

‘My critical skills has [*sic*] improved as [*I*] am now able to act fast/quickly in crisis situations and hence being assertive at all times.’ (P4)

This shows her perception of learning through PoE development.

The skills, experience and knowledge that the participants in this study acquired boosted their self-esteem, confidence and critical thinking skills. One participant in FG2 said that:

‘reflection on what was explained in the classroom assisted him during self-directed learning, as he had to carry out much reflection to assess whether he was doing the right thing or not.’ (P1)

Other participants in FG1 and FG3 also highlighted this issue.

Medley and Horne (2005:31) postulated that use of simulation technology allows undergraduate students to gain and improve skills in a safe, non-threatening, and experiential environment that also provides opportunities for decision making, critical thinking, and team building. According to Harder (2009:23), the purpose of using simulation in health care is the preparation of students for clinical situations they may encounter. Simulation attempts should create as realistic an environment as possible. FG1 participants reported that making use of simulated patients in the clinical skills laboratories boosted their confidence, self-esteem and ethical behaviour, and made them much more aware of the effects of their practice:

‘Making use of the simulated patients took away the feeling of fear and reality kicking in’, reflected a participant from FG1.’ (P3)

According to the participants in this study, completing the required documentation for self-directed practice and recording of bookings at the skills laboratories for the PoE went well. For some of them compilation of the PoE was a great teaching and learning strategy, because learning gaps were identified, especially during self-directed learning in the clinical skills laboratories. However, some participants did not integrate reflective practice adequately because of limited time for compiling a PoE.

## Ethics considerations

Permission to conduct the study was obtained from the Senate Higher Degrees Committee and the School of Nursing at a university in the Western Cape (Project Registration Number 14/9/42). Participants who had volunteered to be included received information about the study, including their right to withdraw from the study at any stage without prejudice. Confidentiality and anonymity was ensured. Participants were put at ease to express their perceptions about the subject of interest. The discussions were recorded with the permission of the students.

### Trustworthiness

The researcher ensured the trustworthiness of the qualitative data through the process of credibility, dependability, and confirmability. According to Burns and Grove (2011:75), rigour in qualitative research is valued because of rigorous studies are seen to be more credible and of greater worth. Credibility was ensured because the researcher constantly verified the data with the participants through paraphrasing what was said. This technique further strengthened the relationship of trust between the researcher and the participants.

To ensure dependability, the FGDs were recorded using a good-quality audio recorder to ensure that the data were captured audibly. The researcher ensured that the FGDs produced in-depth information. The clear description of the research methodology ensures the dependability of this study. An audit trail was used to determine whether the conclusions, interpretations and recommendations could be traced to the data source. Field notes and quotations from the participants were included in the written report of the study.

## Conclusion

Reflective practice enabled the participants to provide the information presented in this article. The findings of this study include descriptions by participants of their experiences in compiling a PoE, and indicate that the process of compiling a PoE requires substantial commitment and responsibility from the student as well as the educators on campus and at the clinical facilities. Some participants expressed the perception that their experiences and confidence, skills (including organisation and record-keeping) and professionalism had been enhanced, whilst others managed to complete their time sheets and progress reports for their PoEs despite some of the PNs displaying a negative attitude to the process.

### Possible limitations

This study was limited to fourth-year nursing students only. The small sample size might have affected the transferability of the findings. However, the FGDs provided a thorough understanding of the perceptions of fourth-year nursing students on reflective practice.
